# Interaction between *Flavivirus* and Cytoskeleton during Virus Replication

**DOI:** 10.1155/2015/427814

**Published:** 2015-08-10

**Authors:** Kar Yue Foo, Hui-Yee Chee

**Affiliations:** Department of Medical Microbiology and Parasitology, Faculty of Medicine and Health Sciences, Universiti Putra Malaysia, 43400 Serdang, Selangor, Malaysia

## Abstract

Flaviviruses are potentially human pathogens that cause major epidemics worldwide. *Flavivirus* interacts with host cell factors to form a favourable virus replication site. Cell cytoskeletons have been observed to have close contact with flaviviruses, which expands the understanding of cytoskeleton functions during virus replication, although many detailed mechanisms are still unclear. The interactions between the virus and host cytoskeletons such as actin filaments, microtubules, and intermediate filaments have provided insight into molecular alterations during the virus infection, such as viral entry, in-cell transport, scaffold assembly, and egress. This review article focuses on the utilization of cytoskeleton by *Flavivirus* and the respective functions during virus replication.

## 1. Introduction

The genus* Flavivirus*, a member of the family Flaviviridae, comprises more than 70 viruses, including dengue virus (DENV), West Nile virus (WNV), tick-borne encephalitis virus (TBEV), Japanese encephalitis virus (JEV), Murray Valley encephalitis virus (MVEV), St. Louis encephalitis virus (SLEV), yellow fever virus (YFV), and many more [1]. Most of the flaviviruses are human pathogens that cause large epidemics globally, resulting in thousands of deaths annually [2]. Dengue virus is an emerging* Flavivirus* that causes major epidemics worldwide [3].* Flavivirus* genome is a single-stranded, positive-sense, ~11 kb RNA genome with a single open reading frame that is directly translated into a polyprotein precursor [4]. The virus particles have two surface viral proteins: the E (envelope) glycoprotein, which is the major determining antigen and is involved in binding and fusion during viral entry, and the M (membrane) protein, which is part of precursor prM, formed during the maturation of virus particle [4]. Cytoskeletons in cells had already been observed to have close contact with virus particles, and virus yields were affected by the disruption of the cytoskeleton [5–8]. Over the years, there were studies using viruses as tools to understand not only the interaction between virus and cytoskeleton but also the cytoskeleton elements of the host cell during virus entry, in-cell transport, process of intracellular assembly, and release of the virus [8–19]. An increased number of dengue cases have been encouraging researchers to study the unclear mechanism causing severe dengue infection in which cytoskeleton disorganisation was observed [20–22]. In this short review, the interaction between* Flavivirus* and cytoskeleton will be discussed.

## 2. Cytoskeleton Characteristics

Cytoskeleton is an interconnected network composed of filamentous polymers and regulatory proteins that enables a cell to prevent cell distortion, organises intracellular organelles, associates cell to external environment, enables shape alterations during movement, and assists intracellular cargo transportation [23]. The cytoskeleton has essential functions that are vital for cellular activity, for example, regulating the branching process of epithelial cells [24]. There are three main types of cytoskeleton: actin filaments, microtubules, and intermediate filaments. First, actin filaments, also known as microfilaments, are the most abundant polymer found in a cell. Actin filaments are packed in bundles or form a network, and both structures help support the cell plasma membrane, forming the cell shape [25]. This globular polymer functions to maintain cell shape, division, migration, junction formation, and intracellular vesicle trafficking [26]. At the end of a polymerising actin filament, the process may aid the escape of viruses from the host cell and infect a new host [25]. The structures that associate with actin filaments for cell contraction are from the myosin protein family [23]. Next are microtubules that are built by tubulin subunit, and it seems that microtubules saturate the entire cytosol [27]. This polar polymer has almost similar functions as actin, including the positioning of organelles and ordered vesicle transport powered by motor proteins [28]. Microtubules associate with molecular motors from the dynein and kinesin family, which function to assemble microtubules during interphase and mitosis cycle and carry cargo for intracellular transportation along the microtubule [23]. Lastly, intermediate filament consists of vimentins, keratins, desmin, peripherin, glial fibrillary acidic protein, neurofilaments, and lamins that have different expressions in tissue or cell type [29]. Intermediate filaments can be presumed to be functioning mainly to strengthen and organise cells together, forming tissue based on the organisation and association with the plasma membrane [29]. Vimentin is important for supporting the structure in the cytoplasm and nucleus, which may cause fragility of cell if disrupted [30]. Vimentin filament strengthens when interacting with microtubules and microfilaments which are assisted by multiple intermediate filament-associated proteins [31, 32]. Vimentin is expressed the most in many cells such as leukocytes, epithelial cells, endothelial cells, and mesenchymal cells [29]. Since intermediate filament is a nonpolar polymer unlike actin and microtubule, it is not involved in cell motility, and no known motor proteins are found along the intermediate filament [23, 29]. These three cytoskeleton polymers are arranged as a network that aids to organise intracellular components and is able to resist or rearrange when force is applied to the cell. The differences between these polymers that differentiate the structure and functions of their network are the dynamic assembly, polarity, and associating motor proteins [23].

## 3. The Involvement of Cytoskeleton in *Flavivirus* Infection

Rearrangement of host cell cytoskeletal and membrane compartments due to activities of virus particles may lead to conformational changes of the cell, known as cytopathic effect, which is a hallmark of virus infection in tissue culture [12, 13]. These alterations vary for different viruses and cell types [10]. The Kunjin virus protein was first known to be associated with cytoskeleton, which was reported to be microtubules [33]. Since then, numerous studies reported* Flavivirus* having close interaction with cytoskeleton elements in the replication cycles of the viruses [8, 9, 14, 15, 18–21, 33–42]. Actin filament was shown to contribute in the entry, production, and release of DENV 2 via interaction with DENV E protein [18, 21]. In addition, a reduction in viral replication of DENV and WNV was observed by blocking the actin function [34, 43]; WNV infection in a mouse model showed proteins associated with actin cytoskeleton, and Rho-GTPase signalling pathways were modified when WNV invaded mice neurons and led to the rearrangement of actin [44]. Rho-GTPase involvement in virus movement and activities has already been described where it controls the assembly of actin and tubulins in neurons [45, 46]. Microtubules [35, 40] and vimentin [14, 42] had been shown to interact directly with* Flavivirus* protein during virus replication. The utilisation of cytoskeleton components may not be direct in some flaviviruses infected cells. For example, actin involved in clathrin-mediated endocytosis (CME) pathway in WNV [36], microtubule-destabilizing protein STMN1 [47], and vimentin rearrangement induced during dengue infection [48].

### 3.1. Entry

Flaviviruses utilise different cytoskeletons for similar activities in host cells. Besides interrupting actin network during viral entry, DENV 2 also utilises and reorganises microtubules and vimentin during the course of infection [9, 21, 48]. However, the involvement of microtubules is suggested to have little to no effect on DENV 2 entrance into ECV304 cells when clarification was made using demecolcine, nocodazole, and paclitaxel drugs that are able to disrupt normal activity of microtubules [9]. JEV internalisation of host neuronal cells uses clathrin-independent mechanism where actin rearrangement takes place when JEV binds to neuronal cells for viral entry [37]. However clathrin-dependent pathway was observed when JEV infects fibroblast cells [37] and Vero cells [49]. On the other hand, WNV internalisation event into the plasma membrane of uninfected cells was studied and shown to be prevented by actin microfilament agitation using cytochalasin D to disrupt actin filaments [36]. Other than utilising cytoskeletons directly, viruses also make use of cell membrane microdomains, such as lipid raft, to enter host cells as seen in DENV [50]. Microtubules, intermediate filaments, and cytoskeletal proteins are the few lipid raft associated proteins, and this perchance indicates secondary involvement of cytoskeletons during the internalisation of viruses exploiting lipid rafts [51]. Similarly, WNV was reported to exploit lipid rafts by interacting with either receptors or accessory proteins in the lipid rafts to promote virus internalisation into host cells [52]. Polymerization of actin was seen to be involved in the CME pathway by coupling with the plasma membrane, making an invaginated path especially for virus internalisation [53–55]. The function of clathrin in facilitating virus entry into host cells has been well documented and, by disrupting clathrin, viral entry was interrupted [36, 49, 56–58]. In addition, the microtubule-associated protein dynamin II was found to aid DENV 2 internalisation through associating with DENV E protein [38]. Taken together, it is not necessary that* Flavivirus* requires the direct utilisation of cytoskeleton for entry to the host cells as the virus may indirectly go through intermediate proteins such as lipid rafts, clathrin, and dynamin II.

### 3.2. In-Cell Transport

Microtubules and their associating proteins play important roles in trafficking viral particles into the host cells, including their responsibility as a transport tool for virions in virus-infected cells [59]. Since microtubules serve as a bidirectional pathway between cell organelles and the viral polyprotein of JEV, it is deduced that the interaction of the viral polyprotein with the microtubules leads to in-cell transport of the proteins from endoplasmic reticulum to Golgi apparatus during JEV replication [35]. Dynein motors in microtubules were studied intensively, and it has been suggested that viral capsids utilise this dynein transport system to move around and exit the cell [60]. DENV, JEV, and WNV were tested to interact with the light chain of dynein (Tctex-1) by binding to its membrane protein. Silencing of Tctex-1 using siRNA method indicated Tctex-1 is involved in the trafficking of these flaviviruses inside the host cell. However, when the same method was tested for YFV and TBV, there were no similar interactions found [39]. Dynein was seen to be utilised by DENV 2 E protein where newly synthesised E proteins were suggested to be trafficked to the assembly site and dissociate from dynein motor as the infection continues. The importance of dynein utilisation during DENV 2 E protein in-cell trafficking was investigated by the disruption of dynein motor activity by dynamitin which subsequently affects the expression of DENV 2 E and C protein [38]. Similarly, microtubule was reported to transport internalised WNV from endosomes to lysosomes [36].

### 3.3. In-Cell Scaffold Assembly

Viral particles require cytoskeletons for movement inside vesicles or in the cytoplasm during assembly to budding compartment [61]. Microtubules were perceived to assist WNV envelope and capsid proteins transported from the perinuclear region to plasma membranes, probably for assembly before releasing virus particles, and disruption of microtubules could markedly reduce virus titres [8]. Host cell cytoskeleton is proposed to play an important role in TBEV maturation process as microtubulin is observed to dense up in the assembly area of TBEV proteins [62]. Another research proposed that TBEV causes reorganization of actin in rat astrocyte instead of microtubules [15]. However, the proper function of cytoskeleton for TBEV is still not fully understood. In DENV-infected cells, vimentin reorganisation was seen where a cage of vimentin is formed, and it has been shown that vimentin is involved in the assembly of DENV 2 particles [9, 16]. Acrylamide, which is able to ruin the organisation of vimentin intermediate filaments [63], caused a reduction of colocalisation of DENV 2 antigen in host cell cytoplasm [9]. The tubulin of DENV 2-infected cells was also suggested to assist in the scaffold assembly of the virus with interactions with the DENV 2 E protein [40]. Cytoskeleton rearrangement induced by the Kunjin virus was suggested to aggregate fibres in the infected cells as there was an increase in vacuole numbers in the cytoplasm, which contains whorls of fibres; when infection progresses, clusters of matured Kunjin virus particle inside the vacuoles in the cytoplasm are possibly released [64].

### 3.4. Egress

WNV was previously illustrated to interact with filopodia, which is composed of a bundle of actin filaments, during the budding process at the plasma membrane of host cells [17]. Kunjin virus yield was apparently slightly reduced when cytochalasin B drug disrupted the organisation of actin filaments, and this event suggested the possibility that any form of disruption could have affected the egress of virus particles [65]. Myosin is an actin motor that was previously reported to be involved in the process of DENV 2 infection, and redistribution of myosin causes reduction of DENV particles being released [41]. A 43 kDa actin protein was identified specifically bound to recombinant domain III of DENV 2 E protein to assist viral particle egress from the host cell [18]. DENV NS1 protein was demonstrated to have a strong association with vimentin, which plays a crucial role in virus replication as well as in the emergence of dengue virus [42]. Recently, microtubule has been shown to be involved in the release of mature JEV [66]. However, drugs that can interfere with the polymerization of the microtubule, such as demecolcine, nocodazole, or paclitaxel, were observed to enhance the production of DENV 2 [9]. The discrepancies seen between different flaviviruses need further investigation. In fact, not many studies were reported on the utilisation of cytoskeleton during the release of* Flavivirus* particles; therefore, mechanisms are still poorly understood.

### 3.5. Other Utilisation of Cytoskeleton

It is shown that during DENV infection the NS4A protein interacts with vimentin where it localizes the protein and assists the RNA replication process [14]. NS3 and NS5 proteins of* Flavivirus* were studied using yeast two-hybrid assay that is able to screen for interacting proteins, and results indicated that NS3 or NS5 protein interacts with many major cellular proteins such as vimentin and myosin, but no further studies were done to identify the mechanism of these interactions [67]. Tight junction protein occludin was displaced to the cytoplasm and actin filament was reorganised when DENV infects host cells [22]. Tight junction proteins are connected to actin filaments [68–70] and the contractile machinery of actin-myosin in vascular endothelial cells is highly regulated [71–73]. Actin-myosin contraction will weaken endothelial cell adhesion and cause vascular permeability [74]. The alteration of tight junctions and actin filament arrangement seen in DENV-infected cells may be one of the causes of endothelial permeability which lead to plasma leakage in DHF patients [12].

## 4. Examples of Cytoskeleton Involvement with Other Viruses

*Flavivirus* is not the only one that utilises cytoskeleton; in fact, many viruses other than* Flavivirus* will either hijack or directly interact with cytoskeletal transport [75]. Here are two examples of viruses that were studied intensively on actin cytoskeleton interactions with respective viruses: influenza A virus (IAV) and respiratory syncytial virus (RSV). Cytoskeleton was observed to be involved in regulating polymorphonuclear leukocyte (PMNL) activities such as neutrophil chemotactic, oxidative, and secretory functions; and because previous report showed that IAV affects PMNL by modifying these cell activities, studies were performed to determine whether IAV alters PMNL cytoskeletons [76–78]. By using flow cytometry, IAV was observed to increase F-actin after 20 minutes of incubation and stimulation by a chemotactic fMet-Leu-Phe (FMLP) on IAV-incubated PMNC, further increasing the level of actin. In addition, alteration on the assembly of polymerized actin was observed when lamellipod/uropod polarity experiment was conducted [78]. Another example is RSV connection with cytoskeleton where actin crosslinking protein filamin A was observed to colocalise with RSV in host cells using a yeast two-hybrid screen. The role of filamin A protein in RSV was studied using fusion protein cytoplasmic tail (FCT), which is critical for virus filamentous assembly by knocking down the protein gene expression using lentivirus-expressing shRNA against filamin A and inhibiting the expression of filamin A. The data showed that filamin A was not necessarily needed in the assembly of RSV [79]. Those studies performed on IAV and RSV with actin cytoskeleton are yet to be performed in* Flavivirus*, but perhaps it could be done in the near future for further understanding of the interactions with cytoskeleton.

## 5. Conclusions

Cytoskeletons, especially actin filament, microtubulins, and intermediate filaments of the host cells, have been observed to be involved with most, if not all, flaviviruses during entry, assembly, and egress processes. Over the years, the observations of the roles of cytoskeletons in viruses-infected cells have developed and improved. These related results from viruses other than* Flavivirus* may indicate that almost all flaviviruses could have a similar method of invading the cells and assembly or evading the host cells. The major challenge is to explicate how viruses adjust the regulation of cytoskeleton motor activity to fulfill their own transport needs. Although the host cells also depend on the cytoskeleton motors for certain cell functions, viruses might interact with them through unique sites, which may have not yet been discovered. The interaction of flaviviruses with cytoskeleton protein binding sites during virus life cycle in various types of host cells will provide extra understanding of these binding sites and expand findings on antiviral production. Since there is still no vaccine for viruses such as DENV and WNV, these detailed studies might be able to provide an insight into producing antivirals.
